# Factors influencing childhood anaemia in Bangladesh: a two level logistic regression analysis

**DOI:** 10.1186/s12887-019-1581-9

**Published:** 2019-06-29

**Authors:** Abu Yusuf, A. S. M. A. Mamun, Md. Kamruzzaman, Aik Saw, Nagah M. Abo El-fetoh, Pete E. Lestrel, Golam Hussain

**Affiliations:** 1 Health Research Group, Department of Statistics, University of Rajshahi, Rajshahi, 6205 Bangladesh; 2 National Orthopaedic Centre of Excellence for Research and Learning (NOCERAL), Department of Orthopaedic Surgery, University of Malaya, Kuala Lumpur, Malaysia; 3 Department of Community Medicine, Faculty of Medicine, Sohag University, Sohag, Egypt; 4 Sections of Orthodontics and Oral Biology, School of Dentistry, University of California, Los Angeles, California USA

**Keywords:** Anemia, Pre-school children, Bangladesh, Prevalence, Multilevel regression

## Abstract

**Background:**

Anemia is not only a major public health problem among children in developing countries, it is also an important predictor for their future growth and development. The objective of this study was to identify possible factors associated with anemia among pre-school children in Bangladesh after removing a cluster effect of the population, and to determine the prevalence of this condition.

**Methods:**

Data for this study was extracted from the 2011 Bangladesh Demographic and Health Survey (BDHS-2011). In this survey, data was collected using a two-stage stratified cluster sampling approach. The chi-square test and a two-level logistic regression model were used for further analysis.

**Results:**

Data from 2231 children aged 6–59 months were included for analysis. The prevalence of child anemia was noted to be 52.10%. Among these anemic children, 48.40% where from urban environment and 53.90% were from rural areas. The prevalence of mild, moderate and severe anemia among children was 57.10, 41.40 and 1.50% respectively. The two-level logistic regression model revealed that the following factors were associated with childhood anemia: children of anemic mothers (*p* < 0.01), undernourished children (*p* < 0.05), younger children (age < 2 years) (*p* < 0.01) and children from poor family (*p* < 0.05). Lastly, anemia was more common among children living in Barisal and Rangpur divisions compared to those from Dhaka division (*p* < 0.01), and among non-Muslims than Muslim (*p* < 0.05).

**Conclusions:**

Our study showed that prevalence of anemia among pre-school children in Bangladesh was very high (52.10%). We noted that young children of anemic mothers, from poor families, and being undernourished were at higher risk of developing anemia. Since most of these risk factors were related to socioeconomic conditions, they were potentially modifiable. Therefore, our findings may be useful for the health authorities to identify children at risk for remedial action and to plan for preventive measures.

## Background

Anemia in children is a serious public health problem especially in developing countries [1]. The global prevalence of anemia among children under 5 years of age is about 43% [2]. It may adversely influence the mental development, physical growth and social behavior in young children [3–6]. In older children, it may be associated with negative behavior, poor school performance and low work capacity [7]. Blood hemoglobin (Hb) level is most common diagnostic test for anemia; in children under 5 years old, anemia is diagnosed when the Hb level is below 11.0 g/dl [8].

In Bangladesh, there has been a noticeable reduction in the mortality rate of mothers and children under 5 years of age following the implementation of Millennium Development Goals (MDGs) [9]. Under the Sustainable Development Goals (SDGs), Bangladesh government has set a target to reduce the mortality rate of under-five children to less than 25 per 1000 live births by 2030 [10]. To ensure continuous improvement in the general health status of the children especially the pre-school group, it is necessary to investigate risk factors that are associated with anemia among them.

A recent study reported that more than 41% of ever-married non-pregnant Bangladeshi women in the reproductive age group had anemia, and the rate was higher among those who were uneducated and under-nourished [11]. Another study on Bangladeshi women reported that low body mass index (BMI) was associated with anemia [12]. For Bangladeshi children, there were a few studies reporting the rate of anemia among those staying in the rural areas [13, 14]. Faruque et al. [13] studied the prevalence of anemia and vitamin A deficiency among Bangladeshi children aged between 2 to 6 in the rural area. Another researcher reported the incidence of anemia among pre-school children in Dhaka city [15]. More recently, Khan et al. [16] conducted a study on anemia among Bangladeshi pre-school children using data which was collected by Bangladesh Demographic and Health Survey (BDHS). In BDHS, data was collected from individuals (household) and they were nested in enumeration areas (clusters). Individuals can be considered as level one, which was the lower level, while clusters were considered as level two. The sample population was big since this was a study at a national level. The sampling areas (clusters, enumeration areas) were selected from different regions in Bangladesh, such as remote rural, rural, semi urban, slam and urban areas [17]. However, cluster effect may potentially influence the finding generated from this dataset. Without removing this cluster effect, the entire analysis might yield misleading results [18].

The aim of this study was to investigate the prevalence of anemia in children, as well as effect of demographic, socio-economic, and parental behavior in the pre-school children population of Bangladesh after removing the clustering effect. We used dataset from BDHS 2011 for analysis.

## Methods

The data were extracted from the Bangladesh Demographic and Health Survey (BDHS-2011). The BDHS-2011 survey collected data from selected households from all over Bangladesh, and the information would represent the population of this country. Socio-economic, demographic, health and lifestyle information were collected from selected subjects [17] from July 2011 to December 27, 2011. BDHS-2011 also collected blood samples from women of reproductive age and pre-school children age between 6 to 59 months to screen anemia. In addition, height and weight of the women and the children were also measured to assess their nutritional status. Basic information related to sampling technique, survey design, survey instruments, measuring system, subject consent, ethics statement, quality control have been described in previous publication [17]. The presence of outliers in the dataset was checked by authors of this study. After removing unusual and missing data, the dataset was reduced to 2231 for analysis.

### Sampling

The BDHS-2011 survey used a two-stage stratified cluster sampling technique to select the households. In the first stage, 600 geographical enumeration areas (EAs) (207 from urban and 393 from rural regions) were selected using random sampling with proportional allocation. In the second stage, 30 households were selected from each EA using systematic sampling. A total of 18,000 ever-married women from 18,000 residential households from the whole country were identified for interview. In addition, one-third of the selected households were considered as sub-samples for blood collection from women and their pre-school children (6–59 months) to screen for anemia.

### Variable

Anemia status of pre-school children was the primary outcome variable in this study. The HemoCue rapid testing method was used in the 2011 BDHS survey to measure Hb levels [17]. The sample was first classified into two groups: anemic children (Hb level < 11.0 g/dl) and non-anemic children (Hb level ≥ 11.0 g/dl). Anemic children were then subdivided into three subgroups based on severity of the condition: mild anemia (Hb level 10.0 to 10.9 g/dl), moderate anemia (Hb level 7.0 to 9.9 g/dl) and severe anemia (Hb level < 7.0 g/dl) [17]. Selected socio-economic and demographic factors were considered as independent variables for the analysis and described in Table 1.

**Table 1:** Description of sleeted outcome and independent variables

Variables	Group	Code	Variables	Group	Code
Children anemia status	Anemic children	1	Religion	Non-Muslim	0
	Non-anemic children	0		Muslim	1
Administrative division	Dhaka	1	Mother’s anemia level	Non-anemic	0
	Chittagong	2		Anemic	1
	Barisal	3	Place of birth/delivery	Home	1
	Khulna	4		Hospital/Clinic	2
	Rajshahi	5	Children’s sex	Male	1
	Rangpur	6		Female	2
	Sylhet	7	Children’s birth weight	Low birth weight	1
Place of residence	Urban	1		Average	2
	Rural	2		Larger than average	3
Mother’s education level	None	0	Children’s age group	Age < 2 year	0
	Primary	1		2 ≤ age < 3 year	1
	Secondary	2		3 ≤ age < 4 year	2
	Higher	3		4 ≤ age < 5 year	3
Maternal age	Age ≤ 20 years	0	Total number of children born	1–2 children	1
	21 ≤ age ≤ 29 years	1		3–5 children	2
	30 ≤ age ≤ 39 years	2		6 and more children	3
	Age ≥ 40 years	3	Number of family members in the household	Member≤4	0
Toilet facilities	Unhygienic	1		Member 5–10	1
	Hygienic	2		Member≥11	2
House hold wealth index	Poor	0	Nutritional status of child	Under-weight	1
	Middle	1		Normal weight	2
	Rich	2		Over-weight	3
				Obese	4

### Statistical analysis

The association between anemia status of pre-school children and their parents’ socio-economic, demographic and behavioral variables were calculated using the Chi-square (χ^2^) test. Variables that were noted to be significantly associated with anemia would be used as independent variables and further analyzed using the multilevel logistic models. Since the data was derived from several different hierarchal levels, the outcome measurements might not be accurate due to cluster effect. A single-level statistical model would not be sufficient for removing this cluster effect [18]. Therefore, we applied a two-level multiple logistic regression analysis to study the association between the diagnoses of anemia and selected the variables. We used the anemia status as the dependent variable. Multilevel logistic regression model is a powerful statistical tool for removing the cluster effect, and for detecting associations between the dependent variable (category) and the independent variables at different levels of the data hierarchy. This model is particularly appropriate for research designs where data for participants are organized at more than one level. In this study, level I was considered for individuals and level II the clusters (EAs). Multicollinearity problem among the independent variables were checked with the standard error (SE). If the value of SE was less than 0.5, we would consider there was multicollinearity problem [19]. Whether the multilevel regression model was appropriate or not was checked by using the median odds ratio (MOR). The value of MOR is always greater than or equal to 1, if MOR = 1, it means there is no cluster variation, but if MOR> 1 there is cluster variation of the dependent variable then it is essential to apply a multilevel regression approach [20]. Statistical significance was determined at a *p* < 0.05 level. Statistical analyses were carried out using STATA (version 13) and SPSS software (version IBM 20).

## Results

**Fig. 1:**
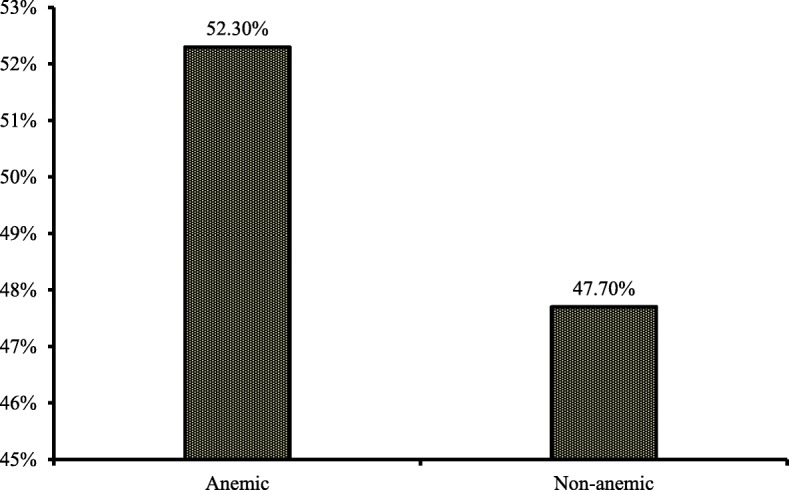
Prevalence of anemia among under five children in Bangladesh

**Fig. 2:**
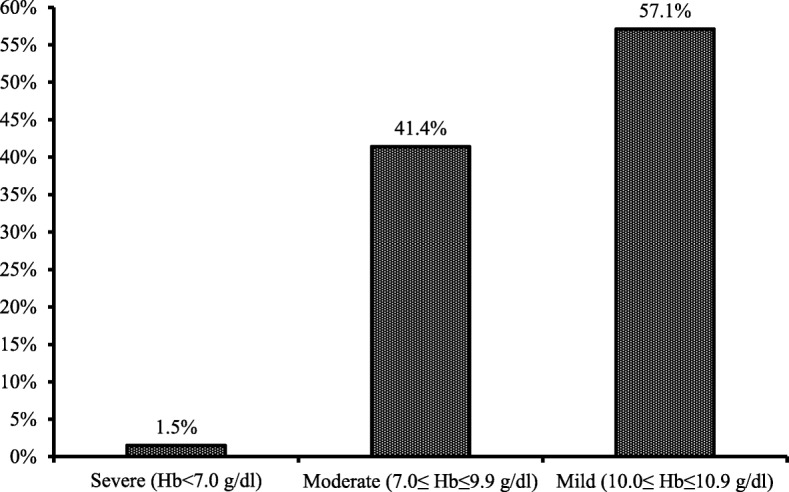
Category of under five anemic children in Bangladesh

The total of 2231 pre-school children (6–59 months) was selected as subjects in this study. We noted that the prevalence of anemia among these children was 52.3% (Fig. 1). When we look at the severity of anemia, we noted that more than 50% of the Bangladeshi pre-school anemic children had mild anemia. More than 40% of them had moderate anemia, and less than 2 % (1.5%) had severe anemia (Fig. 2). The percentage of anemic children was higher rural area (53.9%) compared to those from the urban environment (48.4%). Child anemia more common among boys (53.1%) than girls (51.2%) (Table 2).

**Table 2:** Association between socio-economic, demographic factors and child anemia status in Bangladesh

Variables	Coefficients	SE	z-value	*p*-value	AOR	95% CI for AOR	
						Lower	Upper
Mother anemia level							
No Vs Yes^R^	−0.655	0.087	−6.24	0.001	0.519	0.438	0.616
Child nutritional status							
Normal or over nutrition Vs Under nutrition^R^	−0.197	0.119	−1.66	0.048	0.821	0.650	1.037
Child age group (year)							
2 ≤ age < 3 Vs Age < 2^R^	−0.979	0.139	−7.02	0.002	0.376	0.286	0.494
3 ≤ age < 4 Vs Age < 2^R^	−1.226	0.135	−9.06	0.003	0.294	0.225	0.383
4 ≤ age < 5 Vs Age < 2^R^	−1.472	0.142	−10.39	0.004	0.229	0.174	0.303
Household wealth index							
Middle Vs Poor^R^	−0.286	0.154	−1.86	0.043	0.751	0.555	1.016
Rich Vs Poor^R^	−0.389	0.172	−2.27	0.023	0.678	0.484	0.948
Division							
Chittagong Vs Dhaka^R^	0.233	0.159	1.46	0.144	1.262	0.924	1.724
Barisal Vs Dhaka^R^	0.562	0.184	3.05	0.002	1.755	1.222	2.518
Khulna Vs Dhaka^R^	0.334	0.177	1.88	0.061	1.396	0.985	1.977
Rajshahi Vs Dhaka^R^	0.087	0.175	0.50	0.619	1.090	0.774	1.537
Rangpur Vs Dhaka^R^	0.527	0.171	3.08	0.002	1.694	1.211	2.367
Sylhet Vs Dhaka^R^	0.057	0.166	0.35	0.729	1.059	0.765	1.466
Religion							
Muslim Vs Non-Muslim^R^	−0.388	0.169	−2.29	0.022	0.782	0.487	0. 946
Hosmer and Lemeshow test		Chi-square value = 4.23			df = 9	*p*-value = 0.836	

N.B.: *R* reference case, *AOR* Adjusted Odds ratio, *SE* Standard error, *CI* Confidence interval, *df* degree of freedom

### Chi-square test (χ^2^-test)

The χ^2^-test demonstrated that geographical location (divisions), place of residence, mother’s education level, maternal age, religion, household wealth index, children’s age, toilet facilities, child nutritional status and mother’s anemia status were significantly associated with anemia of the children (Table 1).

These factors were subsequently analyzed as independent variables in the two-level logistic regression model. The value of median odds ratio (MOR) for children with anemia was 1.293, which indicated that the severity of children anemia level was varied among the clusters (enumeration areas). Multilevel logistic regression was, thus, appropriate for analyzing the BDHS-2011 dataset.

### Two-level logistic regression analysis

We observed that the values of standard error (SE) for the selected variables were less than 0.5, which indicated that there was no evidence of multicollinearity among the independent variables in the multilevel logistic regression models. The results of two-level logistic model were interpreted by the adjusted odd ratio (AOR) with a 95% confidence interval (CI), and the corresponding *p*-value. Table 2 shows only the statistically significant influencing factors for child anemia. After removing the cluster effects, the two-level logistic regression model showed that pre-school children from mothers with anemia were more likely to have anemia than those from non-anemic mothers [AOR =0.519, 95% CI: 0.438–0.616; *p* < 0.01]. Undernourished children were at greater risk to have anemia than normal weight or over nourished children [AOR = 0.821, 95% CI: 0.650–0.992; *p* < 0.05]. We also noted that younger children (age < 2 years) had a higher chance to be anemic compared to children in older age groups; age 2 ≤ age < 3 years [AOR = 0.376, 95% CI: 0.286–0.494; *p* < 0.01], age group 3 ≤ age < 4 years [AOR =0.294, 95% CI: 0.225–0.383; *p* < 0.01], and 4 ≤ age < 5 years [AOR = 0.224, 95% CI: 0.174–0.303; *p* < 0.01]. Pre-school children from poor families were more likely to be anemic than those from rich [AOR = 0.678, 95% CI: 0.480–0.948; *p* < 0.05] or middle class [AOR =0.751, 95% CI: 0.555–0.988; *p* < 0.05] families. Children living in the Dhaka administrative division were less likely to be anemic compared to those living in the Barisal division [AOR =1.755, 95% CI: 1.222–2.518; *p* < 0.01] or the Rangpur division [AOR =1.694, 95% CI: 1.211–2.369; *p* < 0.01]. We also noted that children from non-Muslim families were more likely to be anemic than those from Muslim families [AOR =0.782, 95% CI: 0.487–0.946; *p* < 0.05]. Hosmer and Lemeshow test demonstrated that our selected two-level binary logistic model was good fitted to our data (Table 3).

**Table 3:** Effect of socio-economic and demographic factors on anemia among pre-school children in Bangladesh

Variable, Group (*N*, %)	Children anemia			
	No, *N* (%) 1065(47.7)	Yes, *N* (%) 1166 (52.3)	Chi-square value	*p*-value
Division				
Barisal (242, 10.85)	97 (40.1)	145 (59.9)	16.063	0.013
Chittagong (419, 18.78)	201 (48.0)	218 (52.0)		
Dhaka (367, 16.45)	191 (52.0)	176 (48.0)		
Khulna (254, 11.38)	116 (45.7)	138 (54.3)		
Rajshahi (272, 12.20)	138 (50.7)	134 (49.3)		
Rangpur (303, 13.58)	128 (42.2)	175 (57.8)		
Sylhet (374, 16.76)	194 (51.9)	180 (48.1)		
Place of residence				
Urban (672, 30.12)	347 (51.6)	325 (48.4)	5.864	0.015
Rural (1559, 69.88)	718 (46.1)	841 (53.9)		
Mothers’ education				
No education (424, 19.00)	197 (46.5)	227 (53.5)	14.329	0.002
Primary (727, 32.25)	328 (45.1)	399 (54.9)		
Secondary (926, 41.50)	445 (48.1)	481 (51.9)		
Higher (154, 6.90)	95 (61.7)	59 (38.3)		
Children’s sex				
Male (1144, 51.28%)	536 (46.9)	608 (53.1)	0.837	0.360
Female (1087, 48.72%)	530 (48.8)	557 (51.2)		
Maternal age (year)				
Age ≤ 20 (402, 18.02)	148 (36.8)	254 (63.2)	23.797	0.001
21 ≤ Age ≤ 29 (1265, 56.70)	630 (49.8)	635 (50.2)		
30 ≤ Age ≤ 39 (500, 22.41)	256 (51.2)	244 (48.8)		
Age ≥ 40 (64, 2.87)	31 (48.4)	33 (51.6)		
Total number of children				
1–2 children (1311, 58.77)	618 (47.1)	693 (52.9)	1.567	0.457
3–5 children (787, 32.27)	388 (49.3)	399 (50.7)		
6 and more children (133, 5.96)	59 (44.4)	74 (55.6)		
Religion				
Muslim (2017, 90.40)	983 (48.7)	1034 (51.3)	8.417	0.004
Non-Muslim (214, 9.60)	82 (38.3)	132 (61.7)		
Household wealth index				
Poor (952, 42.67)	392 (41.2)	560 (58.8)	34.772	0.001
Middle (402, 18.02)	191 (47.5)	211 (52.5)		
Rich (877, 39.30)	482 (55.0)	395 (45.0)		
Mothers’ anemia status				
Non-anemic (1273, 57.06)	696 (54.7)	577 (45.3)	57.191	0.001
Anemic (958, 42.95)	369 (38.5)	589 (61.5)		
Place of delivery				
Home (1669, 74.81)	784 (47.0)	885 (53.0)	1.543	0.214
Hospital/Clinic (562, 25.19)	281 (50.0)	281 (50.0)		
Children’s birth weight				
Low Birth weight (392, 17.57)	187 (47.7)	205 (52.3)	0.223	0.894
Average (1521,68.17)	730 (48.0)	791 (52.0)		
Larger than average (318, 14.25)	148 (46.5)	170 (53.5)		
Children’s age (year)				
Age < 2 (732, 32.81)	212 (29.0)	520 (71.0)	164.437	0.001
2 ≤ age < 3 (450, 20.17)	231 (51.3)	219 (48.7)		
3 ≤ age < 4 (536, 24.03)	305 (56.9)	231 (43.1)		
4 ≤ age < 5 (513, 22.91)	317 (61.8)	196 (38.2)		
Family members				
≤ 4 (654, 29.31)	314 (46.0)	340 (52.0)	0.055	0.973
5–10 (1390, 62.30)	663 (47.7)	727 (52.3)		
11 ≥ (187, 8.38)	88 (47.1)	99 (52.9)		
Toilet facilities				
Hygiene (1163, 52.13)	593 (51.0)	570 (49.0)	10.301	0.001
Unhygienic (1068, 47.87)	472 (44.2)	596 (55.8)		
Children’s nutritional status				
Under weight (910, 40.79)	419 (46.0)	491 (54.0)	24.816	0.001
Normal (1230, 55.13)	696 (56.6)	534 (43.4)		
Over weight (41, 1.84)	25 (61.0)	16 (39.0)		
Obese (50, 2.24)	30 (60.0)	20 (40.0)		

## Discussion

The prevalence of anemia among pre-school children in Bangladesh was noted to be 52.10% (rural 53.70% and urban 51.70%). This was considerably higher than the global prevalence of anemia of 24.8% [2]. WHO consider child anemia as one of the severe public health problem with a prevalence of greater than 40% in Bangladesh [2]. Higher prevalence rates of child anemia have been observed in other countries such as Indonesia (58.7%) [21], Benin (82%), Mali (83%) [22] and Ghana (78.4%) [23]. However, the prevalence rate in Bangladesh is higher those of neighboring countries Pakistan (33.2%) [24] and India (31.4%) [25] located in the South Asia region. It is also higher than other developing nations like Haiti (38.8%) [26] and Brazil (32.8%) [27].

Most previous studies on child anemia among pre-school children in Bangladesh were limited to the rural areas [13–15]. There was a published study on anemia in pre-school children in Bangladesh that was based nationally representative hierarchy structural dataset (BDHS dataset) [16]. The study applied a single level logistic regression analysis to determine the effects of socio-economic and demographic factors on child anemia. In our opinion, the use of single level statistical model is not appropriate for analyzing this type of nested dataset [18]. We feel that a multilevel regression models should be preferable, and this model has been used for several other studies that were based on BDHS datasets [9, 17, 28, 29]. In this study, two-level logistic regression models were used to determine the effects of socio-economic, demographic, and behavioral factors with anemia among pre-school children in Bangladesh. Moreover, the value of median odds ratio (MOR) was found to be 1.293, indicating that there was a variation anemia status among the 600 enumeration areas (geographical clusters).

This study showed that under-nourished children were more likely to have anemia compared to normal or over-nourished child. This is expected as anemia is one of the clinical and investigation indicators for mal-nourishment. Previous study that was based on BDHS-2011 dataset reported that prevalence of stunted, wasted and underweight children under age 5 was 41, 16 and 36% respectively, and that most of these children were from poor family environment [17]. We also observed that wealth index was an important predictor of child anemia, there was increasing tendency to have anemia with increasing wealth index of the families. We would expect that children from poor family were more likely to be under-nourished. These results were in agreement with other studies from Mali [22] and Indonesia [21]. Our study also showed that the anemic mother was an important risk factor for child anemia, and this has been reported in other studies from Pakistani [24] and Haiti [26]. In Bangladesh, 42% of ever-married women age 15–49 were found to be anemic, with most of them living in poor family environments [17].

Our study noted that age was another factor that was associated with anemia. Young children have limited body reserve, and would be more dependent on their parents for adequate nutrition from daily food intake. Studies on children in Pakistani [24], Haiti [26] and Brazil [27] reported that younger children were more vulnerable to anemia compared to older children. Our study also demonstrated that children living in the Dhaka division were less likely to be anemia than children in other divisions such as Barisal and Rangpur. Being the capital of the nation, the general standard of living would be better since more people staying here would be from the higher wealth quintile compared to those living in the other geographical divisions of the country [17].

The results of the multivariate logistic models in the Khan et al. study [16] that was based on the same dataset showed that water source, wealth index, maternal anemia, age of the children, stunting and division were risk factors for anemia in Bangladeshi pre-school children. When we analyze the data after removing the cluster effect, we were able to identify nutrition level of the child and religion as additional risk factors for this condition.

Most of the factors associated with anemia among pre-school children in Bangladesh were related to poverty. Over the decade, family wealth quintiles, childhood nutrition status and school attendance rate in Bangladesh have generally improved. The level of stunting among children under age 5 has declined from 51% in 2004 to 36% in 2014, while the level of underweight has declined from 43% in 2004 to 33% in 2014 [30]. The authorities should focus on improving the nutritional status both children and mothers, and also eradication of poverty. Based on the observation that prevalence of anemia was highest in children below age of 2 years old, remedial measures should target young children to prevent irreversible adverse effect on the growth and development of these children. We do not have subsequent information on anemia to compare since blood test was not part of the variable in the subsequent BDHS 2014.

### Limitation of this study

Since this study was based on secondary data, we were not able to investigate all factors that may be relevant to anemia in children, including eating habits, parasite infestations, previous hospitalization, availability of filtered water, use of nutritional supplements and gestational birth weight. Prospective study focusing on more specific and relevant variables would be able to generate more useful information.

Despite general improvements of various nutritional and health parameters over the last few years, we were not able to provide any information in the trend since blood Hb level was not part of subsequent BDHS 2014. Blood Hb level is less likely to be influenced by short term alterations in the external environments, therefore trend of change would better reflect the nutritional or health status of the children.

## Conclusion

The prevalence of anemia among pre-school children in Bangladesh was 52.30%. Using a two-level logistic regression model to remove the cluster effect, we found that undernourished children, anemic mothers, younger children (age < 2 years), children from poor family, and non-Muslims were more prone to develop anemia in this country. These findings would be useful for the government to identify children at risk for remedial action, and to prepare preventive measures for this important subgroup of the population.

## Data Availability

The BDHS-2011 datasets are freely available at https://dhsprogram.com/data/dataset/Bangladesh_Standard-DHS_2011.cfm?flag=0

## Abbreviations

AOR: Adjusted Odds Ratio
BDHS: Bangladesh Demographic and Health Survey
CI: Confidence Interval
EA: Enumeration Area
g/dl: Grams Per Decilitre
Hb: Hemoglobin
IBM: International Business Machines
MOR: Median Odds Ratio
NIPORT: National Institute of Population Research and Training
SE: Standard Error
SPSS: Statistical Package for the Social Science Software
WHO: World Health Organization
χ^2^: Chi-Square
