# Correction to: Factors influencing childhood anaemia in Bangladesh: a two level logistic regression analysis

**DOI:** 10.1186/s12887-019-1607-3

**Published:** 2019-07-26

**Authors:** Abu Yusuf, A. S. M. A. Mamun, Md. Kamruzzaman, Aik Saw, Nagah M. Abo El-fetoh, Pete E. Lestrel, Md. Golam Hossain

**Affiliations:** 1 Health Research Group, Department of Statistics, University of Rajshahi, Rajshahi, 6205 Bangladesh; 2 National Orthopaedic Centre of Excellence for Research and Learning (NOCERAL), Department of Orthopaedic Surgery, University of Malaya, Kuala Lumpur, Malaysia; 3 Department of Community Medicine, Faculty of Medicine, Sohag University, Sohag, Egypt; 4 Sections of Orthodontics and Oral Biology, School of Dentistry, University of California, Los Angeles, California USA

**Correction to: BMC Pediatr**

**https://doi.org/10.1186/s12887-019-1581-9**

Following publication of the original article [1], the authors reported that one name in the published online version was incorrect - Golam Hussain should have been Md. Golam Hossain. The corrected name is now provided in the author group section above and the name has been corrected in the original article.
